# A Sustainable Building Promotes Pro-Environmental Behavior: An Observational Study on Food Disposal

**DOI:** 10.1371/journal.pone.0053856

**Published:** 2013-01-09

**Authors:** David W.–L. Wu, Alessandra DiGiacomo, Alan Kingstone

**Affiliations:** 1 Department of Psychology, University of British Columbia, Vancouver, Canada; University of Milan, Italy

## Abstract

In order to develop a more sustainable society, the wider public will need to increase engagement in pro-environmental behaviors. Psychological research on pro-environmental behaviors has thus far focused on identifying individual factors that promote such behavior, designing interventions based on these factors, and evaluating these interventions. Contextual factors that may also influence behavior at an aggregate level have been largely ignored. In the current study, we test a novel hypothesis – whether simply being in a sustainable building can elicit environmentally sustainable behavior. We find support for our hypothesis: people are significantly more likely to correctly choose the proper disposal bin (garbage, compost, recycling) in a building designed with sustainability in mind compared to a building that was not. Questionnaires reveal that these results are not due to self-selection biases. Our study provides empirical support that one's surroundings can have a profound and positive impact on behavior. It also suggests the opportunity for a new line of research that bridges psychology, design, and policy-making in an attempt to understand how the human environment can be designed and used as a subtle yet powerful tool to encourage and achieve aggregate pro-environmental behavior.

## Introduction

Given the current environmental crisis, substantial changes in human behavior will be needed in order to transition into a sustainable society [1]. It has been argued that transmission of cultural norms is essential in producing adaptive human behaviors [2], [3]. How to rapidly dissuade existing unsustainable norms with sustainable ones, not only at an individual level but at a collective level, remains an immense challenge to policy-makers. How these norms can be imbued in the context of one's environment has been largely unexplored as a tool to alter behavior at an aggregate level. Existing laboratory research [4], and applied research in health [5], education [6], and organizational [7] and consumer [8] behavior have converged on the notion that modifying contextual factors may be an effective way to shape behavior. These results raise the exciting possibility that context can serve as a means to promote environmentally conscious behavior outside the lab and in everyday life. The present study examines this possibility.

Despite a substantial need for a revamped approach in psychological research towards sustainable development [1], [9], [10] this field still largely focuses on the individual norms, values, and traits that may promote pro-environmental behavior [11]–[14]. Very few studies address the impact that one's surrounding can have on pro-environmental behavior [15], [16]. While a small handful of studies suggest that subjective user experience is enhanced when occupants move into green buildings [17], [18], there are no published studies that test if the design of a sustainable building can have a positive influence on pro-environmental behavior of a transient population within that space. In the present investigation, we examined whether merely being in a sustainable building might promote pro-environmental behavior – specifically food disposal behavior.

Sustainable buildings, like the Centre for Interactive Research on Sustainability (CIRS) at the University of British Columbia, are becoming increasingly prevalent. Not only is CIRS one of the leading regenerative buildings [19] in North America, it also actively and intentionally embodies and promotes a message of sustainability. In design literature, CIRS would be referred to as having been *designed with intent*, with the hope that the building itself encourages behavioral change [20]. The café at CIRS employs both constraining (e.g., no bottled drinks are available for purchase and all utensils are compostable) and suggestive approaches (e.g., persuasive signs which explain where the food comes from) which could be shaping and influencing user behavior. While these approaches have been explored in theory, there have been little to no evaluation on how effective such approaches are at actually shaping behavior [20], [21].

From the perspective of evaluating the effects of contextual factors on behavior, CIRS provides the perfect environment to test the hypothesis – that being in an environmentally conscious surrounding can elicit environmentally conscious behavior. We chose to observe peoples' food disposal habits because this action involves a decision not constrained by the building itself (i.e., people have to make a decision about where to throw their items). We observed whether people threw out their waste into the correct disposal bin or an incorrect disposal bin during lunch hours across several weeks. As a comparison, we also observed people dispose of waste in the eating area at the Student Union Building (SUB), a building that was not designed with sustainability in mind although importantly it has comparable categories of disposal bins.

## Results and Discussion

**Figure 1:**
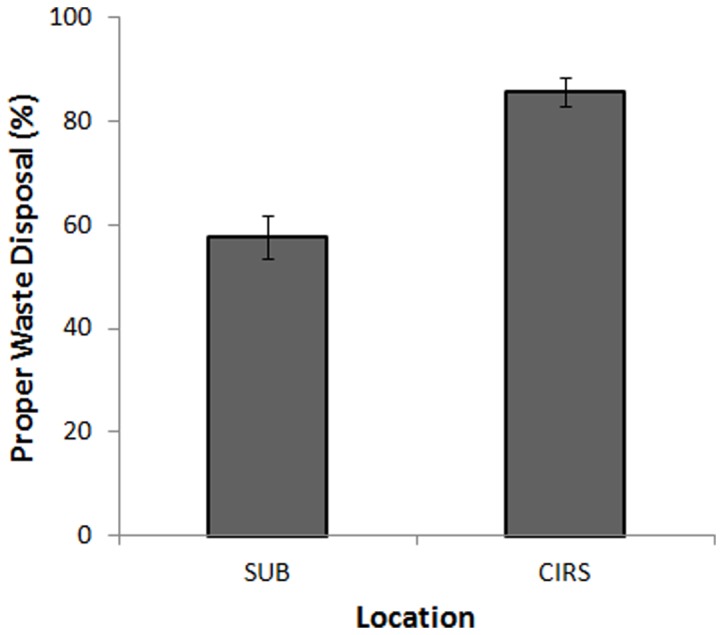
Percentage of proper waste disposal at the SUB or CIRS. People at CIRS (N = 113) were significantly better at properly disposing waste, *M* = 86%, compared to people at the SUB (N = 116), *M* = 58%. Error bars represent standard error of means.

Both sites provided clearly labeled disposal areas, with bins for compost, garbage, and recycling. We observed that people in CIRS were significantly more likely to select the proper disposal unit, *t*(227)  = −5.60, *p*<.001 (see Figure 1). Essentially all cases of improper disposal in both buildings resulted from people incorrectly disposing compostable and recycling material in the garbage. This suggests that placing items in the garbage was the default disposal behavior, regardless of location site, with the critical distinction being that CIRS patrons were more cognizant and deliberate about their bin of choice. Breaking an acquired norm, like the habitual behavior of throwing items in the garbage bin, requires overriding existing automatic responses [22]. The context at CIRS appears to be a powerful tool for achieving that behavioral control.

It should be noted that our reported result is likely a conservative estimate, as extra bins that were found in only one location were not under our observation. For example, a substantial number of people used a stand-alone garbage bin next to the signed compost, garbage, and recycling group of bins under observation in the SUB. At CIRS, there was a paper recycling bin and another compost bin by the coffee area that was not under observation. Thus our results were not due the difference in availability of bins, and we expect the gap between ‘real’ proper disposal rates between CIRS and the SUB to be much greater than reported here if the availability of bins was taken into account.

One outstanding concern is whether our results reflect a sampling bias, i.e., our observation at CIRS could have been derived from people who work daily in CIRS and who already have a higher affinity to behave in a pro-environmental manner. We considered this before our study began, but thought it unlikely as CIRS does not have its own department or student body, and the large lecture theatre (capacity exceeding 400) is used by a broad range of first-year students at the university. A questionnaire conducted in both CIRS and the SUB (N = 61) confirmed this. Only one individual (3%) worked in CIRS, and only one student was in environmental science or a related field (3%). A majority of CIRS patrons also ate regularly at the SUB (65%). Convenience was stated as the main reason people ate at CIRS (65%), followed by trying somewhere new (16%), avoiding crowds (13%), and quality of food (6%). Only one participant (3%) mentioned sustainability as a reason (and it was mentioned subsidiary to convenience). Respondents in the SUB also gave similar motivations, where convenience was mentioned by an overwhelming majority of patrons as the main reason (93%), followed by quality of food (6%). Based on the demographic and motivational responses of patrons in the two venues, we believe it to be highly unlikely that patrons chose to eat at CIRS due to the message of sustainability CIRS embodies.

**Figure 2:**
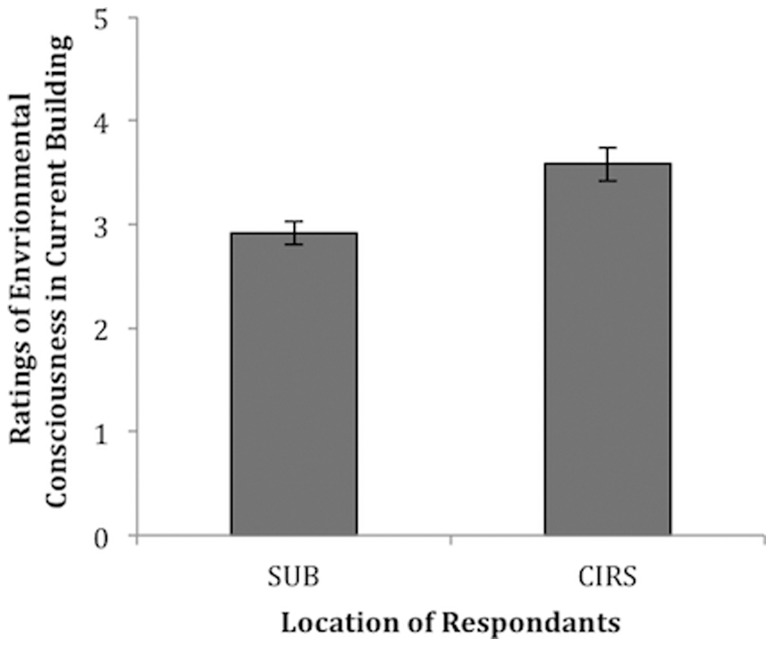
Self-ratings of environmental consciousness of patrons in CIRS and the SUB in their current building. Ratings range from 1 (not at all environmentally conscious) to 5 (extremely environmentally conscious). N = 61, the questionnaire was administered to 31 respondents in CIRS and 30 in the SUB. Respondents in CIRS rated themselves as significantly more environmentally conscious in their current environment (*M* = 3.58, *SD*  = .90) compared to respondents in the SUB (*M* = 2.92, *SD*  = .60). Error bars represent standard error of means.

Why then are patrons at CIRS demonstrating more pro-environmental than at the SUB? It is our speculation that our results may be a large-scale example of an embodied cognition effect [23]. A cornerstone of embodied cognition theory is that cognition is situated, and that real-world contexts actively change how we perceive and act [23], [24]. For example, it has been demonstrated that holding a warm coffee cup or being primed with warmth can induce people to act more warmly towards others [25]. Similarly, we propose that by being in a sustainable context, acting on objects designed for sustainability, and being primed with messages of sustainable induces pro-environmental behavior in CIRS. To explore this theory, we also asked respondents to our questionnaire to rate how environmental conscious they thought they were in in their current building. We found that patrons in CIRS rated themselves significantly higher in environmental consciousness compared to patrons in the SUB, *t*(51)  = 3.41, *p* = .001 (see Figure 2). This result gives preliminary evidence to suggest that the message of sustainability embodied by CIRS may be responsible for the increase in correct disposal behaviour by acting on patron's attitudes towards environmental sustainability. If this is indeed the psychological mechanism behind our results, then there is merit in using priming-based interventions to promote pro-environmental behavior [26]. However, the current study cannot draw a line of causality between building philosophies, to patron attitudes, to patron behaviour. Subsequent controlled experimentation will be required to test this issue.

In sum, our finding suggests that being in an environmentally focused building leads both to feeling and behaving in a more environmentally conscious manner. It is important to note that the purpose of the present investigation was not to find which specific factor or combination of factors at CIRS leads to increased pro-environmental behavior, but rather, to explore whether the pro-environment design that embodies CIRS as a whole would be influential at the level of behavior. Nevertheless, we are currently conducting studies to determine if some specific factors (e.g., the design of the disposal bins [27]) can be manipulated in a manner that modulates pro-environmental behavior in CIRS and in the SUB.

The current study exemplifies the importance of environmentally sustainable developments. Not only are these developments themselves more sustainable in a physical sense, but they influence a large number of users within them to act and think more sustainably as well. These behavioral benefits are powerful and provide exciting opportunities for the future. Though our study raises a number of important questions for future investigation, our hope is that it will also pique the attention of behavioral scientists, designers, engineers, and policy-makers that there is evidence that physical context can have a profound influence on pro-environmental behavior. With urbanization rapidly increasingly globally, the design of new sustainable infrastructure can be a remarkable tool for creating new sustainable norms that may be essential to elicit sustainable behaviors. We believe that intentionality and mindfulness in the design process of sustainable spaces will bring about intentionality and mindfulness in the thoughts and actions of people using the space.

## Materials and Methods

### Ethics statement

This study was done with ethical approval from the University of British Columbia Behavioural Research Ethics Board.

A pair of coders (either D. W.-L. W., A. D., or a research assistant) went to CIRS or the SUB on random days throughout the weeks of February and March, 2012, between 11am and 2pm. Observations were always made on the same disposal bins at the SUB and at CIRS. Coding was done per throw. That is, if a person threw a napkin and a compostable container in one throw in the compost that would mean the person was 100% correct. However, if the person threw out the napkin in the garbage and the container in the compost that would be one incorrect (napkin should have been compost) and one correct (take-out container properly composted) throw, resulting in that person being 50% correct. Coding was done independently and only observations which both coders clearly saw were counted. Inter-rater reliability was high: there was absolute agreement on 86% of observations. We took the average percentage on observations that were not agreed on. If the discrepant observations are discarded, the same results are achieved (SUB: *M* = 58%, CIRS: *M* = 89%). There were 116 observations made in the SUB (43% females), and 113 observations made in CIRS (57% females).

After the behavioral data was collected, patrons of CIRS and the SUB were approached randomly during lunchtime and were asked whether they wanted to answer a few questions about their experience eating at their current venue. A total of 61 responses were collected, 31 from CIRS and 30 from the SUB. Questions were given verbally from a written script, and respondents answered verbally (with the researcher jotting down the responses). General demographics were similar in both locations – students comprised the vast majority of patrons. In CIRS, 5 out of 31 respondents (16%) were staff or faculty, and 24 out of 31 (77%) were students from various disciplines (2 respondents did not fit into either category). In the SUB 2 out of 30 (7%) respondents were staff or faculty, while the remaining 28 respondents (93%) were students from various disciplines.
